# Association between mental health, caries experience and gingival health of adolescents in sub-urban Nigeria

**DOI:** 10.1186/s12903-021-01589-x

**Published:** 2021-04-30

**Authors:** Maha El Tantawi, Morenike Oluwatoyin Folayan, Olakunle Oginni, Abiola Adetokunbo Adeniyi, Boladale Mapayi, Randa Yassin, Nneka M. Chukwumah, Nadia A. Sam-Agudu

**Affiliations:** 1 Department of Pediatric Dentistry and Dental Public Health, Alexandria University, Alexandria, Egypt; 2 Department of Child Dental Health, Obafemi Awolowo University, Ile-Ife, Nigeria; 3 Department of Mental Health, Obafemi Awolowo University, Ile-Ife, Nigeria; 4 Faculty of Dentistry, University of British Columbia, Vancouver, Canada; 5 Department of Preventive Dentistry, School of Dentistry, University of Benin, Benin, Nigeria; 6 International Research Center of Excellence, Institute of Human Virology Nigeria, Abuja, Nigeria; 7 Institute of Human Virology and Department of Pediatrics, University of Maryland School of Medicine, Baltimore, USA

**Keywords:** Adolescents, Caries, Gingivitis, Mental health, Oral diseases, Nigeria

## Abstract

**Background:**

This study assessed the association of mental health problems and risk indicators of mental health problems with caries experience and moderate/severe gingivitis in adolescents.

**Methods:**

A cross-sectional household survey was conducted in Osun State, Nigeria. Data collected from 10 to 19-years-old adolescents between December 2018 and January 2019 were sociodemographic variables (age, sex, socioeconomic status); oral health indicators (tooth brushing, use of fluoridated toothpaste, consumption of refined carbohydrates in-between-meals, dental services utilization, dental anxiety and plaque); mental health indicators (smoking habits, intake of alcohol and use of psychoactive drugs) and mental health problems (low and high). Gingival health (healthy gingiva/mild gingivitis versus moderate/severe gingivitis) and caries experience (present or absent) were also assessed. A series of five logistic regression models were constructed to determine the association between presence of caries experience and presence of moderate/severe gingivitis) with blocks of independent variables. The blocks were: model 1—sociodemographic factors; model 2—oral health indicators; model 3—mental health indicators and model 4—mental health problems. Model 5 included all factors from models 1 to 4.

**Results:**

There were 1234 adolescents with a mean (SD) age of 14.6 (2.7) years. Also, 21.1% of participants had high risk of mental health problems, 3.7% had caries experience, and 8.1% had moderate/severe gingivitis. Model 5 had the best fit for the two dependent variables. The use of psychoactive substances (AOR 2.67; 95% CI 1.14, 6.26) was associated with significantly higher odds of caries experience. The frequent consumption of refined carbohydrates in-between-meals (AOR: 0.41; 95% CI 0.25, 0.66) and severe dental anxiety (AOR0.48; 95% CI 0.23, 0.99) were associated with significantly lower odds of moderate/severe gingivitis. Plaque was associated with significant higher odds of moderate/severe gingivitis (AOR 13.50; 95% CI 8.66, 21.04). High risk of mental health problems was not significantly associated with caries experience (AOR 1.84; 95% CI 0.97, 3.49) or moderate/severe gingivitis (AOR 0.80; 95% CI 0.45, 1.44).

**Conclusion:**

The association between mental problems and risk indicators with oral diseases in Nigerian adolescents indicates a need for integrated mental and oral health care to improve the wellbeing of adolescents.

## Background

Oral health can affect mental health and vice versa [1]. Poor oral health can affect one’s self-esteem and self-confidence, contributing to general anxiety and depression [2, 3]. Conversely, pharmacological management of depression can cause xerostomia, with associated risk of caries and gingival inflammation [4]. Poor oral health in adolescence may have life-long health implications, including increased risk of memory loss, dementia, and Alzheimer’s disease [5]. The low-grade chronic inflammation associated with caries and periodontal diseases and related chronic oxidative stress may induce molecular mimicry that causes autoimmune reactions, ultimately resulting in chronic health disorders such as neurodegenerative diseases [6].

Certain behaviors serve as links between oral and mental health, often causing worsening of one or the other. For example, tobacco smoking, which has been associated with mental health problems [7, 8], induces hyper-inflammation, with systemic changes in inflammatory cytokine levels and immunoglobulins. This can contribute to worsening of chronic inflammatory conditions such as multiple sclerosis, with concomitant deterioration in periodontal health [9]. Conversely, patients with poor mental health (severe mental illness, affective disorders, and eating disorders) often neglect their oral health, and have worse oral health status than otherwise healthy individuals [10].

Mental health problems often begin in adolescence, between 10 and 19 years of age. Approximately half of all mental health conditions start by 14 years of age, with depression being a leading cause of disability and suicide, the third leading cause of death among adolescents [11]. The World Health Organization reports that one in six people with mental health problems are adolescents, and that mental health problems account for 16% of the global burden of disease and injury in this population. When left untreated, mental health problems in adolescence extend to adulthood with negative impact on quality of life [12].

Similar to mental health problems, oral diseases also often begin or worsen in adolescence. Hormonal changes in adolescence increase the risk of gingival inflammation [13]. Adolescence is also a period of increases in sugar intake, cigarette smoking initiation, and orthodontic treatments, which may lead to lifelong oral health issues [14]. However, empirical studies on mental health among individuals with oral diseases, especially in this age, and vice versa are limited.

The aim of the study was to assess the risk for mental health problems and the prevalence of oral disease (caries experience and moderate to severe gingivitis) among 10–19-year-old adolescents in South-West Nigeria. It also seeks to determine the association between mental health problems and oral diseases in the study population. The study’s null hypothesis was that there will be no association between mental health problems and oral diseases.

## Methods

### Study population and study design

Data were collected through a cross-sectional household survey conducted between December 2018 and January 2019 in Ife Central Local Government Area of Osun State, a semi-urban community in South-West Nigeria. Adolescents were eligible to participate in the study if they were between 10 and 19 years old and had parental consent/assent/individual informed consent as appropriate. Adolescents who were critically ill and could not give independent responses to the survey were excluded. Recruitment of participants continued until the required sample size was reached.

### Sample size and sampling technique

Based on the power calculations of the original study [15], the required number of participants was 1234. With this sample size of 1234 in the present study, post-hoc analyses indicated a power of at least 0.90 to detect significant associations between risk for mental health problems and oral health conditions (caries and gingivitis) [16].

We used a multistage sampling strategy to collect data for the primary study [17]. First, 70 of the 700 enumeration areas in Ife Central Local Government Area were sampled using a simple random technique. Next, every other household in the selected enumeration areas was selected as eligible. Finally, in each household, one adolescent meeting the inclusion criteria was recruited for the study. Whenever a household declined to participate or was found ineligible, the next eligible household was substituted.

#### Explanatory variables

##### Age and sex

These were assessed using single questions inquiring about participants’ age in years and their sex at birth, respectively.

##### Socioeconomic status

Socioeconomic status was determined by using an adapted version of the index developed by Olusanya et al. [18], based on data collected in the same setting as our study [19]. This is a multiple-item socio-economic status index combining the mother’s level of education with the father’s occupation. The study participants’ maternal level of education was classified as follows: no formal education, Quranic and primary school education (scored 2); secondary school education (scored 1) and tertiary education (scored 0).

The father’s occupation was also categorized into three levels: civil servants or skilled professionals with a tertiary level of education (scored 1); civil servants or skilled professionals with a secondary level of education (scored 2); unskilled, unemployed, students, and civil servants or skilled professionals with a primary and/or Quranic education (scored 3). The socio-economic class of each participating adolescent was determined by adding the score of the mother’s level of education to that of the father’s occupation. Each adolescent was allocated into social classes I–V (class I, upper class; class II, upper middle class; class III, middle class; class IV, lower middle class; class V, lower class). These were later categorized for analysis into 3 classes by combining I and II into upper class, III into middle class and IV and V into lower class. When an adolescent had lost a parent, their socioeconomic status was determined using the occupation and educational level of the living biological parent. When an adolescent had lost both parents, their socioeconomic status was determined using the occupation and educational level of the guardian [18, 19].

##### Tooth brushing

Respondents were asked about the frequency of tooth brushing using the following alternatives—irregularly or never, once a week, few (2–3) times a week; once a day, and more than once a day. Respondents who chose the options ‘*irregularly or never, once a week, few (2–3) times a week, once a day*’ were classified as with no preventive dental care [20].

##### Use of fluoridated toothpaste

Respondents were asked about the frequency of using fluoridated toothpaste when tooth-brushing using the following options—‘*Always*, *quite often*, *seldom*, *not at all’*. Respondents who chose the options ‘*quite often*, *seldom*, *not at all*’ were classified as not having undertaken preventive dental care [20].

##### Consumption of refined carbohydrates in-between-meals

Respondents were asked about the frequency of consuming sugar-containing snacks or drinks between main meals, using the following options—‘*About 3 times a day or more, about twice a day, about once a day, occasionally; not every day, rarely or never eat between meals*’. Respondents who chose the options ‘*About 3 times a day or more, about twice a day, about once a day*’, were classified as not having undertaken preventive dental care [20].

##### Dental service utilization

Respondents were asked about the time of the last dental check-up using the following options—*within the last 6 months, more than 6 months to one year ago, more than 1–2 years ago, more than 2–5 years ago, more than 5 years, never, do not remember*. Attending at least one dental check-up within the last year was defined as preventive care use. Respondents who chose the options ‘*more than 1–2 years ago, more than 2–5 years ago, more than 5 years, never, do not remember’* were classified as not having undertaken preventive dental care [20].

##### Smoking habits

Information on respondents’ cigarette-smoking was elicited using a single question, with six response options—*‘No, never’, ‘No, I used to, but I quit’, ‘Yes, once a month or less’, ‘Yes, few times (2–3) a month’, ‘Yes, few times (2–3) a week’* and *‘Yes, once a day or more’*. Responses were grouped into smokers, and non-smokers categorizations. Participants who chose options *‘Yes, once a month or less’, ‘Yes, few times (2–3) a month’, ‘Yes, few times (2–3) a week’* and *‘Yes, once a day or more’* were classified as smokers [20].

##### Intake of alcohol

Respondents were asked about their frequency of alcohol intake (*Every day, once a week, less than once a week, never, not sure* and *no response*). The responses were categorized into yes *(Every day, once a week, less than once a week)* and no (never, not sure).

##### Use of psychoactive drugs

Information on the use of psychoactive drugs (marijuana, solvent glue, cocaine, heroin, tramadol, codeine, injecting cocaine or heroin using a syringe and needle) was collected. The responses were dichotomized into ‘0’ when there was no indication of psychoactive substance use, and ‘1’ if there was indication of use of any of the substances.

##### Dental anxiety

Dental anxiety was measured using the Corah dental anxiety scale. The scale has four questions with five-point Likert scale responses. The level of anxiety was assessed as a summation of the scores of all questions. Possible scores range from 4 to a maximum of 20. The scores were coded as follows, 4 to 8 as normal anxiety, 9–12 as moderate anxiety, 13–14 as high anxiety, and 15–20 as severe anxiety bordering on phobia [21]. The scale has been validated for use in Nigeria [20]. The Cronbach’s alpha for this study was 0.92.

##### Oral hygiene

Oral hygiene status was determined using the plaque index of Loe and Silness [22]. Each participant was examined sitting, under natural light, with sterile dental mirrors by trained dentists. The plaque index score was based on six numerical values representing the amount of debris on six index permanent teeth 12, 16, 24, 32, 36, and 44. The mesial, distal, buccal, and lingual gingival areas of the index teeth were scored from 0 (no plaques) to 3 (abundance of soft matter within the gingival pocket and/or on the tooth and gingival margin). The mean score for each tooth was obtained and the mean score for the individual was determined by adding the indices for each tooth and dividing by the number of teeth examined.

#### Outcome variables

##### Mental health problems

Study participants’ risk of mental health problems was assessed using the Global Health Questionnaire-12 (GHQ-12) developed by David Goldberg [23]. It is one of the most widely used tools in screening for mental ill-health among adolescents and adults. The GHQ-12 has been validated for use in Nigeria [24, 25]. Its major value lies in its ability to segregate those who are mentally healthy from those who are at risk of mental health problems. Each item is rated by four possible responses, ‘*Not at all’*, ‘*No more than usual*’, ‘*Rather more than usual*’ and ‘*Much more than usual*’, scored from 0 to 3 respectively. The total possible score on the GHQ-12 ranges from 0 to 36, with cut-off point of ≥ 7 in Nigeria: participants with scores ≥ 7 were categorized as having high levels of mental distress, while those with scores less than 7 were categorized as having low levels of mental distress. In a study by Gureje et al [26], the alpha coefficient of the GHQ-12 was 0.82. The Cronbach’s alpha for this study was 0.80.

##### Gingival health

The severity of gingivitis was evaluated with the gingival index of Löe and Silness [27]. Changes in the gingiva around six index permanent teeth (12, 16, 24, 32, 36, and 44) were assessed. Four areas of each index tooth were scored. The scores were summed and divided by four to give the gingival index for each tooth. The gingival index of each participant was obtained by adding the values of all index teeth and dividing by six. Gingivitis was classified into mild, moderate, or severe, with values of 0.1–1, 1.1–2, and 2.1–3, respectively and dichotomized for analysis into healthy gingiva/mild gingivitis versus moderate/severe gingivitis [28].

##### Caries experience

Assessment of caries experience was performed after oral hygiene and gingival health status were determined. Each participant was examined sitting on a chair under natural day light using dental mirrors. Teeth were cleaned of debris and dried using a sterile gauze before assessment. The presence of caries experience was assessed using the decayed (D), missing (M), and filled (F) teeth (T) index following the World Health Organization criteria at cavitation level [29]. Three calibrated examiners (Kappa statistic between pairs ≥ 0.9) conducted the examination. The DMFT index was obtained by adding the D, M and F scores. Caries experience was further divided into caries experience present (DMFT > 0) or absent (DMFT = 0).

##### Data analysis

Descriptive analysis was performed to determine the proportion of adolescents with each sociodemographic variable (age, sex, socioeconomic status), and oral health indicators (oral hygiene status, dental anxiety, tooth-brushing frequency, use of fluoridated toothpaste, intake of refined carbohydrates in-between meals, and dental services utilization). The proportion of adolescents with mental health problems and mental health risk behaviors (cigarette smoking, intake of alcohol, use of psychoactive substances) were also determined.

Bivariate analyses were conducted to determine the associations between the explanatory variables and the outcome variables (caries experience and moderate to severe gingivitis) using Chi square test (for categorical variables or Fisher exact test when applicable), Mann Whitney U test (for ordinal variables) and t-test for quantitative variables.

We assumed that the two oral diseases (caries experience and moderate to severe gingivitis) are associated with sociodemographic factors, oral health indicators in addition to mental health indicators which may or may not be independent of the effect of mental health problems as another factor associated with oral diseases. To assess this assumption, a series of logistic regression models were constructed, and blocks of factors were introduced in successive models: Model 1 included the sociodemographic factors, model 2 included oral health indicators, model 3 included mental health indicators and model 4 included the risk of mental health problems. Model 5 included all factors from models 1–4. Multicollinearity was checked and correlated variables were removed from the models. The estimated coefficients were expressed as adjusted odds ratios (AOR) and their 95% confidence intervals were calculated. The fit of each model was assessed using Nagelkerke R2 and − 2 log likelihood (− 2LL). Statistical analyses were conducted using IBM SPSS for Windows version 22.0 (IBM Corp., Armonk, N.Y., USA). Statistical significance was inferred at *p* < 0.05.

##### Ethical considerations

Ethical approval was obtained from the Ethics and Research Committee of the Institute of Public Health, Obafemi Awolowo University, Ile-Ife, Nigeria (IPHOAU/12/669). Approval for conduct of the study was obtained from the Local Government Authority prior to commencement. The study was conducted in full compliance with the protocol submitted to the ethics committee and in full accordance with the Helsinki declaration. Informed consent was obtained from one parent of each study participant aged 10–11-years-old prior to enrollment. Both parental consent and participant assent were obtained for those 12–13-years-old. Consent was obtained from study participants and their parents if participants were 14 to 18 years in line with guidance from the Federal Ministry of Health [30]. Consent was obtained from participants who were 19 years old. Efforts were made to minimize risks to participants by ensuring that anonymized data collection was done privately using an electronic data platform. Study participants’ discomfort with the personal nature of questions was limited by ensuring that field workers were trained on how to ask sensitive questions and clarify non-verbal cues observed during the interviews. No compensation was paid for study participation. Participants were each given a gift of a sachet of fluoridated toothpaste valued at less than $1.00.

## Results

Complete data were available for 1234 participants out of the 1469 who were invited to participate (response rate = 84.0%), with mean (SD) age of 14.6 (2.7) years (Table 1). Most participants reported frequent consumption of refined carbohydrates in-between meals (62.4%), no dental visits within the last 12 months (98.9%), less than twice daily toothbrushing (91.9%) and regular use of fluoridated toothpaste when they brushed their teeth (91.1%). The mean plaque index was 0.82. Alcohol intake (12.0%) was more frequent than the use of psychoactive substances (5.8%) and cigarette smoking (1.4%). About a third of participants (35.7%) reported moderate dental anxiety.

**Table 1:** Demographic and oral health profile of adolescents in Ile-Ife, Nigeria, and their association with risk of mental health problems [N = 1234]

Variables	Low risk N = 974 (78.9%) n (%)	High risk N = 260 (21.1%) n (%)	*p* value	Total N = 1234 n (%)
**Age in years**				
Mean (SD)	14.43 (2.63)	15.31 (2.61)	< 0.001*	14.6 (2.7)
**Sex at birth**				
Male	565 (80.9)	133 (19.1)	0.048*	698 (56.6)
Female	409 (76.3)	127 (23.7)		536 (43.4)
**Socio-economic status**				
High	354 (82.9)	73 (17.1)	0.04*	427 (34.6)
Middle	322 (76.3)	100 (23.7)		422 (34.2)
Low	298 (77.4)	87 (22.6)		385 (31.2)
**Frequent consumption of refined carbohydrates in between meals**				
Yes	634 (83.9)	122 (16.1)	< 0.0001*	756 (62.4)
No	324 (71.1)	132 (28.9)		456 (37.6)
**Visit to the dentist within the last 12 months**				
Yes	11 (78.6)	3 (21.4)	1.00	14 (1.1)
No	963 (78.9)	257 (21.1)		1220 (98.9)
**Toothbrushing twice daily**				
Yes	73 (73.0)	27 (27.0)	0.13	100 (8.1)
No	901 (79.5)	233 (20.5)		1134 (91.9)
**Regular use of fluoridated toothpaste**				
Yes	948 (84.3)	176 (15.7)	< 0.0001*	1124 (91.1)
No	26 (23.6)	84 (76.4)		110 (8.9)
**Plaque index score**				
Mean (SD)	0.80 (0.57)	0.91 (0.54)	0.003*	0.82 (0.57)
**Intake of alcohol**				
Yes	105 (70.9)	43 (29.1)	0.01*	148 (12.0)
No	869 (80.0)	217 (20.0)		1086 (88.0)
**Use of psychoactive substances**				
Yes	56 (77.8)	16 (22.2)	0.81	72 (5.8)
No	918 (79.0)	244 (21.0)		1162 (94.2)
**Cigarette smoking**				
Yes	11 (64.7)	6 (35.3)	0.15	17 (1.4)
No	963 (79.1)	254 (20.9)		1217 (98.6)
**Dental anxiety**				
Normal	303 (83.2)	61 (16.8)	0.002*	364 (29.5)
Moderate	357 (81.1)	83 (18.9)		440 (35.7)
High	116 (73.9)	41 (26.1)		157 (12.7)
Severe	198 (72.5)	75 (27.5)		273 (22.1)

^*^Statistically significant at *p*< 0.05

Table 1 also shows that 260 (21.1%) of adolescents had high risk of mental health problems. Adolescents with high risk of mental health problems were significantly older (15.31 versus 14.43 years; *p* < 0.001) and had significantly higher plaque index scores (0.91 versus 0.80; *p *= 0.003) than those at low risk.

Significantly less males than females had high risk of mental health problems (19.1% and 23.7%; *p* = 0.048); and significantly more adolescents with low than high socioeconomic status had high risk of mental health problems (22.6% versus 17.1%; *p* = 0.04). Also, compared to those who regularly used fluoridated toothpaste, significantly more adolescents who did not regularly use fluoridated toothpaste (76.4% versus 15.7%; *p* < 0.001) had high risk of mental health problems, as did more adolescents reporting alcohol intake compared to those reporting no alcohol intake (29.1% versus 20.0%; *p* = 0.01). In addition, adolescents with greater severity of dental anxiety had high risk of mental health problems (*p* < 0.001). Frequent consumption of refined carbohydrates in-between meals was significantly associated with lower risk of mental health problems (16.1% versus 28.9%; *p* < 0.0001).

Table 2 shows that 46 (3.7%) adolescents had caries experience. Participants with caries experience were significantly older than those with no caries experience (15.6 years versus 14.6 years; *p *= 0.01). Significantly more participants using psychoactive drugs had caries experience than those not using these drugs (11.1% versus 3.3%; *p* = 0.001), while more participants with high risk of mental health problems (6.2% versus 3.1%; *p* = 0.02) had caries experience than those with low risk.

**Table 2:** Association between caries experience and moderate to severe gingivitis and demographic and oral and mental health risk indicators in adolescents in Ile-Ife, Nigeria [N = 1234]

Variables	Caries experience			Gingival condition		
	Absent N = 1188 (96.3%) n (%)	Present N = 46 (3.7%) n (%)	*p* value	Healthy/mild gingivitis N = 1134 (91.9%) n (%)	Moderate/severe gingivitis N = 100 (8.1%) n (%)	*p* value
Age: mean (SD)	14.6 (2.6)	15.6 (2.7)	0.01*	14.6 (2.7)	14.7 (2.7)	0.63
**Sex at birth**						
Male	670 (96.0)	28 (4.0)	0.55	637 (91.3)	61 (8.7)	0.35
Female	518 (96.6)	18 (3.4)		497 (92.7)	39 (7.3)	
**Socioeconomic status**						
High	415 (97.2)	12 (2.8)	0.37	396 (92.7)	31 (7.3)	0.63
Middle	406 (96.2)	16 (3.8)		388 (91.9)	34 (8.1)	
Low	367 (95.3)	18 (4.7)		350 (90.9)	35 (9.1)	
**Frequent consumption of refined carbohydrates in-between-meals**						
Yes	727 (96.2)	29 (3.8)	0.92	705 (93.3)	51 (6.7)	0.02*
No	439 (96.3)	17 (3.7)		408 (89.5)	48 (10.5)	
**Plaque Index score**						
Mean (SD)	0.80 (0.54)	0.82 (0.57)	0.86	0.75 (0.51)	1.60 (0.54)	< 0.001*
**Intake of alcohol**						
Yes	141 (95.3)	7 (4.7)	0.49	131 (88.5)	17 (11.5)	0.11
No	1047 (96.4)	39 (3.6)		1003 (92.4)	83 (7.6)	
**Use of psychoactive substances**						
Yes	64 (88.9)	8 (11.1)	0.001*	64 (88.9)	8 (11.1)	0.34
No	1124 (96.7)	38 (3.3)		1070 (92.1)	92 (7.9)	
**Cigarette smoking**						
Yes	16 (94.1)	1 (5.9)	0.48	15 (88.2)	2 (11.8)	0.64
No	1172 (96.3)	45 (3.7)		1119 (91.9)	98 (8.1)	
**Risk for mental health problems**						
Low	944 (96.9)	30 (3.1)	0.02*	896 (92.0)	78 (8.0)	0.81
High	244 (93.8)	16 (6.2)		238 (91.5)	22 (8.5)	
**Dental anxiety**						
Normal	346 (95.1)	18 (4.9)	0.37	322 (88.5)	42 (11.5)	0.01*
Moderate	423 (96.1)	17 (3.9)		404 (91.8)	36 (8.2)	
High	153 (97.5)	4 (2.5)		148 (94.3)	9 (5.7)	
Severe	266 (97.4)	7 (2.6)		260 (95.2)	13 (4.8)	

^*^Statistically significant at *p*< 0.05

Also, 100 (8.1%) participants had moderate to severe gingivitis. The presence of moderate to severe gingivitis was significantly associated with frequent consumption of refined carbohydrates in-between-meals (*p* = 0.02), plaque accumulation (*p* < 0.0001) and dental anxiety (*p* = 0.01). Further exploration showed that among those who frequently consumed refined carbohydrates in-between-meals, 295 (32.8%) had healthy gingiva, 544 (60.5%) had mild gingivitis, 56 (6.2%) had moderate gingivitis and 4 (0.4%) had severe gingivitis. Among those who do not frequently consume refined carbohydrates in-between-meals, 199 (39.3%) had healthy gingiva, 250 (49.3%) had mild gingivitis, 55 (10.8%) had moderate gingivitis and 3 (0.6%) had severe gingivitis. There was statistically significant differences between the groups (*p* < 0.0001).

Table 3 shows that Model 5 had the best fit (− 2LL = 400.34, Nagelkerke R2 = 0.07). Age was associated with significantly higher odds of caries experience presence in model 1 (AOR 1.23; 95% CI 1.09, 1.38) and in model 5 (AOR 1.15; 95% CI 1.02, 1.30). Severe dental anxiety was associated with significantly lower odds of caries experience presence in model 2 (AOR 0.39; 95% CI 0.16, 0.94) but not in model 5 (AOR 0.43; 95% CI 0.18, 1.06). Use of psychoactive substances was associated with significantly higher odds of caries experience presence in model 3 (AOR: 3.70; 95% CI 1.49, 9.15) and in model 5 (AOR 2.67; 95% CI 1.14, 6.26). High risk of mental health problems was associated with significantly higher odds of cares experience presence in model 4 (AOR 1.90; 95% CI 1.04, 3.45) but not in model 5 (AOR 1.84; 95% CI 0.97, 3.49).

**Table 3:** Associations between the presence of caries experience and sociodemographic background, oral and mental health risk indicators in adolescents in Ile-Ife, Nigeria [N = 1234]

Variables	Model 1	Model 2	Model 3	Model 4	Model 5
Age	1.23 (1.09, 1.38)*	–	–	–	1.15 (1.02, 1.30)*
**Sex at birth**					
Male	1.16 (0.65, 2.08)	–	–	–	1.11 (0.61, 2.03)
Female	1.00	–	–	–	1.00
**Socioeconomic status**					
High	0.52 (0.25, 1.07)	–	–	–	0.51 (0.24, 1.07)
Middle	0.75 (0.38, 1.46)	–	–	–	0.69 (0.35, 1.37)
Low	1.00	–	–	–	1.00
**Frequent consumption of refined carbohydrates in-between-meals**					
Yes	–	0.91 (0.50, 1.65)	–	–	0.94 (0.50, 1.76)
No	–	1.00	–	–	1.00
Plaque Index score		0.82 (0.48, 1.39)		–	0.78 (0.45, 1.34)
**Dental anxiety**					
Normal	–	1.00	–	–	1.00
Moderate	–	0.67 (0.35, 1.28)	–	–	0.66 (0.34, 1.27)
High	–	0.41 (0.14, 1.22)	–	–	0.40 (0.13, 1.21)
Severe	–	0.39 (0.16, 0.94)*	–	–	0.43 (0.18, 1.06)
**Intake of alcohol**					
Yes	–	–	0.88 (0.38, 2.06)	–	–
No	–	–	1.00	–	–
**Use of psychoactive substances**					
Yes	–	–	3.70 (1.49, 9.15)*	–	2.67 (1.14, 6.26)*
No	–	–	1.00	–	1.00
**Cigarette smoking**					
Yes	–	–	1.11 (0.22, 5.59)	–	0.99 (0.20, 4.99)
No	–	–	1.00	–	1.00
**Risk for mental health problems**					
Low	–	–	–	1.00	1.00
High	–	–	–	1.90 (1.04, 3.45)*	1.84 (0.97, 3.49)
Nagelkerke’s R2	0.04	0.02	0.02	0.01	0.07
− 2LL	422.38	425.58	420.94	432.23	400.34

^*^Statistically significant at *p*< 0.05; AOR: adjusted odds ratio; CI: confidence interval

Model 1: sociodemographic factors; Model 2: oral health indicators; Model 3: mental health indicators; Model 4: risk of mental health problems; Model 5: all factors included in models 1–4

Intake of alcohol was not included in Model 5 because of collinearity with use of psychoactive substances

Table 4 shows that for assessing the factors associated with moderate to severe gingivitis, the full model, model 5, had the best fit (− 2LL = 530.97, Nagelkerke R2 = 0.34) followed by model 2 (2LL = 587.60, Nagelkerke R2 = 0.34) which included the oral health indicators. The frequent consumption of refined carbohydrates in between meals was associated with significantly lower odds of the presence of moderate to severe gingivitis in model 2 (AOR 0.42; 95% CI 0.27, 0.65) and in model 5 (AOR 0.41; 95% CI 0.25, 0.66). Plaque was significantly associated with higher odds of the presence of moderate to severe gingivitis in Model 2 (AOR 13.23; 95% CI 8.70, 20.14) and in Model 5 (AOR 13.50; 95% CI 8.66, 21.04). Severe dental anxiety was associated with significantly lower odds of the presence of moderate to severe gingivitis in model 5 (AOR 0.48; 95% CI 0.23, 0.99) but not in model 2 (AOR 0.53; 95% CI 0.28, 1.01). The risk of mental health problems was not significantly associated with the presence of moderate to severe gingivitis in model 4 (AOR 1.09; 95% CI 0.71, 1.69) or model 5 (AOR 0.80; 95% CI 0.45, 1.44).

**Table 4:** Associations between the presence of moderate to severe gingivitis and sociodemographic background, oral and mental health risk indicators in adolescents in Ile-Ife, Nigeria [N = 1234]

Variables	Model 1	Model 2	Model 3	Model 4	Model 5
Age	1.03 (0.96, 1.11)	–	–	–	1.03 (0.94, 1.14)
**Sex at birth**					
Male	1.20 (0.82, 1.75)	–	–	–	1.09 (0.68, 1.75)
Female	1.00	–	–	–	1.00
**Socio-economic status**					
High	0.87 (0.55, 1.37)	–	–	–	0.96 (0.53, 1.74)
Middle	0.79 (0.50, 1.25)	–	–	–	0.95 (0.54, 1.67)
Low	1.00	–	–	–	1.00
**Frequent consumption of refined carbohydrates between meals**					
Yes	–	0.42 (0.27, 0.65)*	–	–	0.41 (0.25, 0.66)*
No	–	1.00	–	–	1.00
Plaque Index score		13.23 (8.70, 20.14)*	–	–	13.50 (8.66, 21.04)*
**Dental anxiety**					
Normal		1.00	–	–	1.00
Moderate	–	0.79 (0.48, 1.32)	–	–	0.91 (0.54, 1.55)
High	–	0.65 (0.30, 1.39)	–	–	0.70 (0.331, 1.58)
Severe	–	0.53 (0.28, 1.01)	–	–	0.48 (0.23, 0.99)*
**Intake of alcohol**					
Yes	–	–	1.25 (0.70, 2.22)	–	–
No	–	–	1.00	–	–
**Use of psychoactive substances**					
Yes	–	–	1.31 (0.58, 2.97)	–	–
No	–	–	1.00	–	–
**Cigarette smoking**					
Yes	–	–	0.90 (0.19, 4.30)	–	0.69 (0.11, 4.21)
No	–	–	1.00	–	1.00
**Risk for mental health problems**					
Low	–	–	–	1.00	1.00
High	–	–	–	1.09 (0.71, 1.69)	0.80 (0.45, 1.44)
Nagelkerke’s R2	0.004	0.34	0.003	< 0.0001	0.34
− 2LL	852.47	587.60	745.31	854.90	530.97

Model 1: sociodemographic factors; Model 2: oral health indicators; Model 3: mental health indicators; Model 4: risk of mental health problems; Model 5: all factors included in models 1–4

Intake of alcohol and use of psychoactive substances were not included in Model 5 because of collinearity with cigarette smoking

^*^Statistically significant at *p*< 0.05; AOR: adjusted odds ratio; CI: confidence interval

## Discussion

The study showed that the risk of mental health problems was high and the prevalence of caries experience and moderate/severe gingivitis were low in our adolescent cohort. The risk of mental health problems was not associated with the presence of caries experience in the multivariable regression, while the use of psychoactive substances was associated with higher odds of caries experience. Neither the risk of mental health problems nor mental health risk indicators were associated with the presence of moderate to severe gingivitis. The null hypothesis of the study which focused on mental problems cannot thus, be rejected.

One of the strengths of this study is the data collection methods: the data were collected through a household survey, which ensured that in- and out-of-school adolescents, and adolescents within all socio-economic categories were included. Additionally, the study is a pioneer in examining associations between the use of psychoactive substances, mental health, and oral health specifically among adolescents in Nigeria.

The cross-sectional design, however, limits the use of the finding to infer causality. In particular, the study design is not capable of capturing the dynamic nature of mental health problems and their evolution over time, which is associated with potential for changes in the pattern and strength of associations with oral health practices and clinical outcomes. Furthermore, some underlying factors that may be associated with oral disease and mental health problems, such as sense of coherence [31, 32] and locus of control [33, 34] were not assessed in this study. Partial mouth recording was used to assess gingival inflammation and plaque accumulation in the present study [22, 27] and this may have resulted in under- or over-estimation of oral health conditions. However, these two indices are widely used, and they facilitate comparison with other studies. Another study limitation is the possibility that respondents with mental health problems under-reported their symptoms because of stigmatization. It is therefore important to interpret the study findings within the context of the sample profile. Among other adolescents (or other age groups) with higher levels of oral disease or mental health problems, the observed associations may differ in direction or magnitude. Future studies are needed to confirm the study findings at different levels of disease prevalence and severity, to unravel the associations between mental health problems, caries experience and consumption of refined carbohydrates, and to better understand the reasons for the generally low levels of oral diseases observed in the present study.

The high prevalence of mental distress among adolescents in our cohort should be a source of concern, given that this was a community-based study and the prevalence determined is higher than the prevalence of 9.9% reported amongst 15–18-year-olds at a psychiatric hospital in Nigeria [35]. The difference may be because we assessed the general risk of mental health problems rather than specific mental health problems or diseases, and the fact that we included younger adolescents who may have already started experiencing mental health problems. Mental health problems are a leading cause of disability among young people globally [36], and as indicated in this study, these problems may be associated with oral disease.

The high risk of mental health problems associated with plaque accumulation and alcohol intake may possibly indicate the clustering of high-risk behaviors in vulnerable adolescents. Caries risk indicators to consider among patients at risk of poor mental health are the regular use of fluoridated toothpaste, frequency of consumption of refined carbohydrates in-between-meals, and plaque index scores, all of which were associated with risks of mental health problems in the present study. The study findings suggest the importance of integrated oral and mental care and prevention for vulnerable adolescents. In addition, it is also important to identify shared risk factors that increase the odds of both mental and oral health conditions and their mechanisms of action.

Paradoxically, mental health problems were associated with low frequency of consumption of refined carbohydrates in-between-meals, a key risk factor for caries [37]; and brushing twice daily, a protective factor against caries [38]. These associations should be interpreted within the context of the cross-sectional nature of the study, which is unable to establish time sequence. For example, greater brushing may have occurred after dental pain resulting from oral diseases. In which case, brushing would be a reaction rather than a preventive behavior and thus, more likely to indicate poor oral and mental health. Also, toothbrushing may not necessarily be thorough but the high frequency may be fear-induced, thereby explaining the higher plaque accumulation among study participants with high risk of mental health problems, even with frequent toothbrushing. The general neglect that often follows poor mental health could lead to less consumption of all meals, including refined carbohydrates.

The association between dental anxiety and high risk of mental health problems may suggest that the mental health problems may moderate or mediate the pathway between dental anxiety and caries and interfere with the utilization of dental care services [39]. Longitudinal studies are needed to elucidate these complex associations and assess the connection between mental health problems and the risk of caries. This moderating or mediating pathway is very possible as caries experience was associated with the use of psychoactive substances; and the use of the use of psychoactive substances is associated with mental health problems. The use of psychoactive substances causes a reduction in salivary flow [40] which may lead to caries.

There was no association between mental health problems and risk of moderate-to-severe gingivitis. On the other hand, frequent consumption of refined carbohydrates in-between meals was associated with lower odds of moderate-to-severe gingivitis, and thus seems to have a protective effect. The participants resided in an agro-dependent area, which is associated with a plant-based diet [41]. It is possible that the plant-based diet mitigates the risk of moderate/severe gingivitis associated with greater consumption of refined carbohydrates [42]. However, this specific hypothesis has not been previously investigated, and a comprehensive assessment of dietary intake was not conducted in the present study. We recommend that this study limitation be addressed in future studies. Additionally, most adolescents in our cohort were classified as not having moderate to severe gingivitis but had mild gingivitis rather than healthy gingiva. This mild level of gingivitis may also be affected by frequent consumption of refined carbohydrates in-between meals. Thus, the observed association in the study may indicate greater association between mild gingivitis and frequent consumption of refined carbohydrates in-between meals, rather than suggesting a protective effect of the consumption of refined carbohydrates against moderate to severe gingivitis.

Also, the lower odds of moderate to severe gingivitis in association with severe dental anxiety may reflect the nature of the relationship between dental visits and oral health in Nigeria. Though severe dental anxiety is associated with less dental visits for preventive dental care [43], in Nigeria, dental visits occur mostly when there are dental problems or pain [44]. Thus, not visiting the dentist (whether because of severe dental anxiety or otherwise) may be associated with lower odds having moderate to severe gingivitis.

Our results suggest that inequalities exist in the risk of mental health problems by sex and socioeconomic status, with greater burden among females and those of low socioeconomic status. Our findings contrast with an earlier Nigerian study that indicated no sex differences in the prevalence of mental health disorders among adults 18 years and above [45] though there are sex and socioeconomic disparities in the prevalence of mental health disorders in other parts of the world [46, 47]. Age, sex and socioeconomic status were also risk factors for caries among adolescents in Nigeria in previous studies [48–50]. These shared risk factors between caries and mental health suggest the need for a common risk factor approach to manage oral and mental health among adolescents in Nigeria. Implementation models for such integrated health care delivery systems have previously been proposed [51]. These and other models for integrated management of interlinked high-morbidity diseases affecting adolescents need to be further evaluated for feasibility, impact, and cost-effectiveness.

## Conclusion

Adolescents with high risk of mental health problems may also be at high risk of caries experience although this association is attenuated when other risk indicators are considered. Oral health risk factors that protect against moderate-to-severe gingivitis may also protect from the risks of mental health challenges. The relationship between mental health and oral health in adolescents appears complex, and the pathway for the relationships remains unclear. Larger, prospective studies are needed to decipher the directions and pathways for the associations observed in this study.

## Data Availability

All data generated for this study are presented in the manuscript. Patient-level data can however be accessible on reasonable request from one of the authors, Morenike Oluwatoyin Folayan.
